# Trichoblastoma Arising From the Nevus Sebaceus of Jadassohn

**DOI:** 10.7759/cureus.15325

**Published:** 2021-05-29

**Authors:** Fatimazahra Chahboun, Madiha Eljazouly, Mounia Elomari, Faycal Abbad, Soumiya Chiheb

**Affiliations:** 1 Dermatology Unit, Cheikh Khalifa International University Hospital, Mohammed VI University of Health Sciences, Casablanca, MAR; 2 Plastic and Reconstructive Surgery, Cheikh Khalifa International University Hospital, Mohammed VI University of Health Sciences, Casablanca, MAR; 3 Pathology, Cheikh Khalifa International University Hospital, Mohammed VI University of Health Sciences, Casablanca, MAR

**Keywords:** trichoblastoma, nevus, sebaceous

## Abstract

Trichoblastoma is a rare benign skin adnexal tumour, belonging to the category of trichogenic tumours. The clinical and histological findings may often be confused with basal cell carcinoma, a malignant epidermal skin tumour.

We report here a case of a 70-year-old man presented with a dome-shaped, dark-pigmented nodule within a yellowish hairless plaque on the scalp. The plaque had existed since childhood. However, the central pigmented nodule began appearing three months ago and enlarging gradually. The patient had no medical history. Furthermore the physical examination revealed a translucent, verrucous, and yellowish plaque, with central and pigmented nodule measuring 0.7 × 0.5 cm. Also basal cell carcinoma and trichoblastoma’s diagnosis were discussed. The patient was subsequently referred to the plastic surgery department, where he underwent a total excision. The histological examination was in favour of trichoblastoma arising from the nevus sebaceus. After 24 months of checking, no recurrence was observed.

Trichoblastoma is a benign adnexal tumour. Its progression to malignant trichoblastoma (or trichoblastic carcinoma) is possible, but remains exceptional. Therefore, complete excision should be proposed to the patient at the time of diagnosis.

## Introduction

Trichoblastoma is a rare benign adnexal tumour, belonging to the category of trichogenic tumours. It shows epithelial and mesenchymal cell proliferations that recapitulate the development of the hair follicle [1]. It may occur alone or on top of a sebaceous nevus of Jadassohn. The clinical and histological findings may often be confused with basal cell carcinoma, a malignant epidermal skin tumour. The surgery remains the gold standard in the treatment of this tumour.

We report a case of trichoblastoma arising from a sebaceous Jadassohn's nevus of the scalp.

## Case presentation

**Figure 1:**
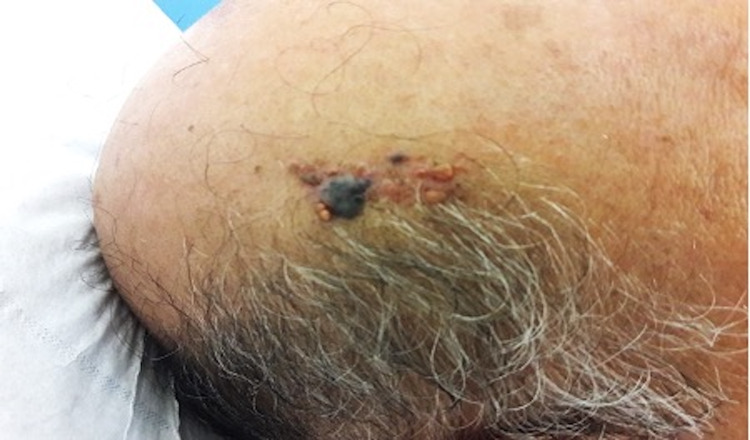
Translucent, verrucous, and yellowish plaque, with central and pigmented nodule measuring 0.7 × 0.5 cm

A 70-year-old man presented with a dome-shaped, dark-pigmented nodule within a yellowish hairless plaque on the scalp. The plaque had existed since childhood. However, the central pigmented nodule began appearing three months ago and enlarging gradually. The patient had no medical history of cutaneous or internal malignancies. Furthermore, the physical examination revealed a translucent, verrucous, and yellowish plaque, with central and pigmented nodule measuring 0.7 × 0.5 cm (Figure 1).

**Figure 2:**
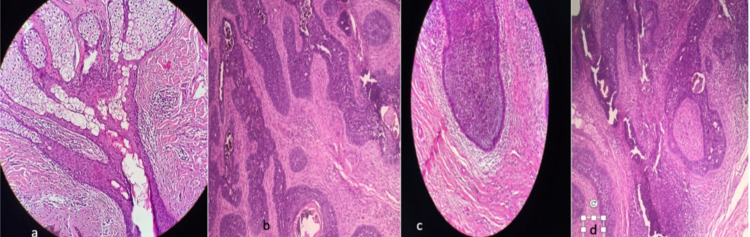
(a) Histological image of a nevus sebaceus. (b,c) Proliferation of primitive and abortive hair follicle structures, arranged in well-defined nests of basal cells with peripheral palisades surrounded by fibrous stroma, no retraction artifact. (d) Histological image with high magnification.

Also, basal cell carcinoma and trichoblastoma’s diagnosis were discussed. The patient was subsequently referred to the plastic surgery department, where he underwent a total excision. The histological examination was in favour of trichoblastoma arising from the nevus sebaceus (Figure 2). After 24 months of checking, no recurrence was observed.

## Discussion

Jadassohn's sebaceous hamartoma (JSH) is a complex congenital dysembryoplasia affecting mainly the scalp and the face. It presents either as a patch of alopecia or as a patch with elevated or warty surface. Numerous tumours can develop on the nevus sebaceous including trichoblastoma, trichoepithelioma and syringocystadenoma papilliferum [2].

In a retrospective study treating Jadassohn's sebaceous nevi, 8.5% of the cases were observed with secondary tumours even though the study contains 450 cases. Syringocystadenoma papilliferum was the most common benign tumour (2.7%), followed by trichoblastoma (1.6%) and trichilemmoma (1.6%), while basal cell carcinoma (0.9%) was the most frequent malignant tumour on sebaceous nevus [2].

Trichoblastoma is an adnexal tumour that differentiates from the primitive hair follicle usually on the face and scalp of middle-aged adults, with no gender preference [3]. It can develop on healthy skin or on a sebaceous hamartoma of Jadassohn. The typical clinical appearance is a single, flesh-coloured or yellowish, well-circumscribed, non-ulcerated tumour that usually progresses in size over several months or years [4,5].

The dermoscopic appearance of trichoblastoma described in the literature is based on small series [6]. The presence of blue-ovoid nests and blue-grey globules is a common sign but not exclusive of basal cell carcinoma than the trichoblastoma. Detection of these dermoscopic features can help the clinician to guide the differential diagnosis [6]. However, definitive and certain diagnosis requires histological examination.

Histological examination of trichoblastoma reveals a basaloid proliferation in which the cells may be arranged in cords, sheets or discrete clusters surrounded by a fibrous stroma. Trichoblastoma can be differentiated histologically from other skin tumours with similar clinical presentations, mainly basal cell carcinoma and trichoepithelioma. However, basaloid cell tumours, which are a natural progression of sebaceous hamartoma and have long been considered basal cell carcinomas, are in fact, in the majority of cases, trichoblastomas. In difficult cases, immunostaining can be used, in particular the follicular stem cell marker PHDLA-1 which is expressed in hair follicle tumours [7].

Furthermore, melanotrichoblastoma, or pigmented trichoblastoma, is a very rare variant of trichoblastoma that should be known and in which the neoplastic epithelium is colonised by melanocytic dendritic cells [8,9].

To date, surgical treatment based on complete excision is the gold standard in the treatment of this tumour [10].

## Conclusions

Trichoblastoma is a benign adnexal tumor. The clinical and histological findings can often be confused with basal cell carcinoma, a malignant epidermal skin tumor. Its progression to malignant trichoblastoma (or trichoblastic carcinoma) is possible, but remains exceptional. Therefore, complete excision should be proposed to the patient at the time of diagnosis.
